# Does the Use of Perennials in Flower Beds Necessarily Imply Sustainability?

**DOI:** 10.3390/plants12244113

**Published:** 2023-12-08

**Authors:** Miroslav Poje, Vesna Židovec, Tatjana Prebeg, Mihael Kušen

**Affiliations:** Department of Ornamental Plants, Landscape Architecture and Garden Art, Faculty of Agriculture, University of Zagreb, Svetošimunska cesta 25, 10000 Zagreb, Croatia

**Keywords:** conventional design, flower bed sustainability index, green spaces, maintenance

## Abstract

Green spaces are becoming increasingly important for cities due to the growing pressures of urbanization and climate change. Along with trees, shrubs, and lawns, flower beds are an important part of urban green spaces. The majority of flower beds in public spaces consist of annual and biennial flower species. Such seasonal flower beds feature eye-catching colors but require significant effort to maintain and manage. Compared to these conventional flower beds, those with herbaceous perennials are more ecologically effective and less costly to maintain, and therefore more sustainable. The aim of this research was to analyze flower beds with perennials in the public green spaces of the city of Zagreb and to develop a tool based on predefined criteria and indicators to evaluate the sustainability of flower beds. In the context of the research, sustainability meant appropriate selection of flower species based on environmental conditions (temperature, light, precipitation), species diversity, greater ground cover and extensiveness of maintenance. The research results showed that there were 327 flower beds with perennials planted in the ground. The constructed Flower Bed Sustainability Index (FBSI) showed that the majority of these perennial beds (56.3%) had a conventional character, as only 28.1% of the beds had a completely correct species selection. This result indicates that the use of perennials does not necessarily guarantee the sustainability of flower beds, since, as in the case of flower beds with seasonal flowers, it depends, among other things, on the correct selection of species adapted to local environmental conditions. The FBSI is shown to be a suitable tool for assessing the degree of sustainability of a flower bed and could be a useful tool in landscape design and management of such types of green spaces.

## 1. Introduction

Green spaces are becoming increasingly important due to their numerous functions [1], but also because of the increasing pressure from urbanization [2,3] and climate change [4,5]. In view of these challenges, their sustainable planning and design is no longer just desirable, but essential.

Although there are different terminologies, definitions and interpretations, two basic approaches to the design of outdoor spaces can be defined: conventional and sustainable. Of course, sustainable design is a general concept most closely associated with Nature-based Solutions (NbS) [6], but it also shares many common principles with ecological design [7,8,9].

Conventional design encompasses long-established practices and principles in the design of outdoor spaces. It is characterized by its esthetic focus and adherence to conventional norms, often ignoring ecological and sustainability principles. This approach tends to favor ornamental and non-native plant species for their visual appeal, even though they may not be adapted to the local environment and therefore cannot provide important ecosystem services or support local wildlife. Conventional solutions are usually very water- and chemical-intensive and focus on maintaining a manicured appearance [10,11]. However, such practices can lead to problems such as excessive water consumption, soil degradation and the decline of native biodiversity. Furthermore, conventional landscaping may not be adequately equipped to address the pressing environmental challenges of our time such as climate change, habitat loss and the need for sustainable resource management. While esthetics play an important role in landscape design, it is becoming increasingly clear that a holistic and ecologically sound approach to designing outdoor spaces that are not only visually appealing but also ecologically sustainable and resilient is essential.

Sustainable landscape design is a holistic and environmentally conscious approach to the design of outdoor spaces that aims to minimize ecological impact while maximizing functionality and long-term viability. It focuses on creating esthetically pleasing landscapes that coexist harmoniously with nature and promote human well-being and ecological health [12,13]. This approach includes a range of strategies, such as selecting native or adaptive plant species, creating habitats for pollinators, using environmentally friendly materials and implementing efficient irrigation systems, all of which aim to reduce resource consumption and minimize the ecological footprint of the designed space. Sustainable landscaping also includes the management of stormwater through techniques such as rain gardens and permeable paving to mitigate the negative impact of urbanization on water quality and quantity. In addition, the use of renewable energy sources is often integrated to reduce energy consumption and minimize greenhouse gas emissions. Overall, sustainable landscaping provides a holistic and future-oriented framework for designing outdoor spaces that not only meet the needs of the present, but also ensure the well-being of future generations while respecting the natural environment.

In order to successfully overcome all the challenges mentioned, ways must be found to manage the financial resources allocated to the design and maintenance of public green spaces as efficiently as possible. Esthetic criteria should still play an important role, but the greatest emphasis should be on functionality in relation to the challenges mentioned above. In addition to the reduced financial resources available for the maintenance of green spaces, there is also a noticeable and increasing lack of professional staff to manage these spaces. This leads to a simplification in the realization of parks and green spaces that are mainly transformed into mowed lawns with trees [14], which does not sufficiently contribute to sustainability and biodiversity. A high-quality design of green spaces should ensure visual quality and complexity with minimal use of resources [15]. In this way, it is possible to create habitats that are self-sustaining to a certain degree and esthetically interesting due to their (bio)diversity [16,17]. This would not only improve the sustainability of the urban landscape, but also help to reduce maintenance costs [18,19].

Alongside trees, shrubs and lawns, flower beds are a common vegetation element in urban landscapes. Although they have a much smaller impact on the urban environment compared to trees, for example, flower beds are one of the vegetation elements that are perceived very positively by citizens. It is therefore somewhat surprising that there are only a relatively small number of research studies on them [20]. In the context of general landscape design and taking into account the characteristics that define flower beds, they can also be divided into conventional and sustainable. Of course, it is important to point out that such a classification of flower beds (this also applies to landscape design as a whole) does not only include the two extremes mentioned, as there is a continuum with many intermediate types in between [21].

The conventional approach to flower bed design usually results in annuals and biennials being planted. The reason for the increased use of seasonal flowering species is that they bloom profusely, have a long flowering period and are characterized by lush colors that have a positive effect on people [22,23]. The choice of flower species is often restricted by a limited palette of plants arranged in groups and/or in a repetitive order, usually with only one species planted [24]. This is justified from an esthetic point of view, as the aim of such flower beds is a simple and harmonious design. Some studies show that citizens prefer this type of flower beds, while they perceive “wild” forms of nature as untidy [25,26,27,28]. On the other hand, this type of flower bed, created within certain rules and shapes, is sometimes perceived as uncreative and monotonous [29]. Although the price of the plant material is relatively low, it is changed seasonally, resulting in significant implementation costs and the need for frequent maintenance, as they are labor-intensive areas that require many resources (fertilization, irrigation, protection against diseases and pests, and the like).

The sustainable flower bed is based on the fundamental principle that it is important to select and place plants in locations that are best suited to their specific needs and characteristics (*right plant*, *right place*), and not just on the basis of esthetic criteria such as color, height, texture or blooming time. This concept is the key to a healthy and thriving flower bed [30]. When selecting flower species for a sustainable flower bed, it is important to consider their tolerance to extreme temperatures and the climate in general, preferred soil type and pH, water requirements, light preferences, resistance to diseases and pests, maintenance needs and other factors. All of the above criteria must be met by both native and non-native plants. The plants selected should be as closely related as possible to their natural habitat to ensure the longevity of the flower bed. Plants adapted to local soils, for example, need less fertilization, while plants adapted to local insects and diseases need fewer pesticides. Understanding the interactions between them and their sociability is extremely important for the successful combination of different plant species in different mixed plantings [31]. Establishing a sustainable bed is more expensive than a conventional bed but becomes more cost-effective in later stages when maintenance costs are much lower, even though maintaining such a bed requires more skilled labor. In addition, maintenance costs can be minimized by involving citizens who are willing to participate in the planting and maintenance of the beds with the support of the municipality, which would provide the planting material and a planting plan [32,33].

One way to improve the sustainability of flower beds and thus of public green spaces, is the wider use of herbaceous perennials [34,35], but not only as monocultures or in block form, as is usually the case today. The use of perennials is a more economical way of landscaping as they require less maintenance. They also have a positive effect on soil quality, as the plant material remains unchanged for years [36] and can be a potential solution for reducing and treating stormwater runoff in urban areas [37]. The advantages of combining perennials lie in the diversity and heterogeneity that are attractive to people [38,39,40]. The use of native flowers is a sustainable alternative to the traditional approach of using plant material that relies heavily on soil characteristics, which are often poor in urban environments [41]. Nevertheless, it is necessary to find the right balance, because when designing outdoor areas, one should not rely exclusively on native species, even if these attract a much greater variety of flower-visiting insects [42], for example, because non-native species also have their advantages [43,44,45].

The objectives of this study were: to determine the relationships between the different characteristics of flower beds with perennials, to create a tool for distinguishing between conventional flower beds with perennials and sustainable ones, and to determine which types of flower beds with perennials planted in the ground are predominant in the City of Zagreb. The working hypothesis of this study was that conventional flower beds with perennials prevail over sustainable flower beds in public green spaces in the City of Zagreb.

## 2. Results

### 2.1. Analysis of Flower Beds with Perennials

During the fieldwork, all 640 sites listed in the database as having perennial flower beds were visited. Through direct observation, it was found that 327 perennial flower beds were planted in the ground, which were further analyzed. In addition, 191 beds in various types of planters and containers were recorded, as well as 133 sites where there were no perennial flower beds. Of the 327 flower beds with perennial plants in the ground, not a single one was recorded in the Brezovica district, while the highest number (60; 18.35%) was found in the Novi Zagreb—istok district (Table 1).

Based on the indicators of proper species selection, 28.1% of the flower beds contained flower species that fully met the environmental conditions required for their successful growth and development. About 70% of the flower beds consisted of four or more species, mainly perennials. In 167 flower beds, less than 50% of the area was covered with flowering species. Complete coverage with ground cover plants was recorded in 5.8% of the beds, while the maximum mulch cover was recorded on three beds. With regard to the degree of maintenance, it was estimated that 14.7% of the flowerbeds are not maintained at all, while 7.6% are maintained intensively and 77.7% extensively (Table 2).

The proper selection of species was assessed on the basis of three indicators: resistance to low temperatures, light requirements and the amount of precipitation required. In this study, a maximum positive correlation was found between the three indicators mentioned, i.e., for each flower bed analyzed, the same value was found for all three indicators. However, it should be noted that these are three different indicators and the correct selection of flower species based on one indicator does not necessarily mean the correct selection based on the other indicators.

### 2.2. The Difference between the Area of Flower Beds and the Level of Maintenance Required

The statistical significance of the difference in the average flower bed area between unmaintained, intensively maintained and extensively maintained flower beds was tested using the Kruskal–Wallis test. According to the test results, a statistically significant difference in average mean rank was found where larger flower beds were intensively maintained, while smaller beds were maintained extensively or not maintained at all (Table 3).

### 2.3. The Relationships between the Nine Selected Indicators

The chi-square test was used to test the mutual relationship of nine indicators. To avoid thin cells, certain variables were recoded when performing the chi-square test so that certain categories of individual variables were grouped together. The tests performed revealed certain tendencies between the indicators.

The fewer other types of flowers there were in the beds (or the more perennials there were), the more extensive the maintenance was (χ^2^_(1)_ = 14.614, *p* = 0.000). In other words, the more annual and/or biennial flower species there were, the more intensive the maintenance was (Table 4).

When the flower species were well selected based on their resistance to low temperatures, their light requirements and the amount of precipitation required, the coverage of the bed area was greater, while a less good selection of flower species based on these three indicators resulted in a lower coverage of the bed area (χ^2^_(1)_ = 8.225, *p* = 0.004) (Table 5).

The better the selection according to all three indicators with regard to the correct choice of flower species, the more extensive the maintenance (χ^2^_(1)_ = 3.338, *p* = 0. 043). In other words, the lower the suitability of the plants selected on the basis of the environmental conditions, the more intensive the maintenance (Table 6).

### 2.4. Latent Dimensions of Flowerbed Characteristics

The main factors of the flower beds with perennials were identified by applying the PCA procedure to the indicators representing the characteristics of the flower beds. For transparency of the displayed results, small saturations, i.e., those less than 0.30, were omitted. In the initial factorization of the measurement instrument, in an attempt to determine the most appropriate factor structure, further analysis excluded items whose loadings on the latent factors were less than 0.60 (Presence of mulch and Maintenance level). Consequently, 2 items were omitted, and 7 items were retained for further analysis. Three independent latent dimensions were extracted that explained approximately 86.17% of the total data variance (Table 7).

The first factor, which explains 42.45% of the variance, can be interpreted as “proper selection of flower species”, which is related to the proper selection of plants based on their resistance to low temperatures, light requirements and preferred precipitation levels. Although all three indicators have identical saturation values, they represent three different indicators of correct selection. The maximum correlation is a consequence of the ordinal measurement scale, which has led to a low discriminatory power of the measurement tool.

The second factor is saturated by the following two indicators: coverage with flower species and coverage with ground covers. It can be interpreted as “ground coverage” and is intended to measure the degree of ground cover within the flower bed, regardless of whether it is covered by flower species or ground-cover plants. This factor explains 24.46% of the variance.

The third latent dimension is saturated by two items: the number of flower species and the number of species that are not perennials. Based on the content of the items, the third extracted factor can be interpreted as “flower species diversity”. The negative saturation of the second item (number of flower species) is in line with the original theoretical assumptions: the more other flower categories (such as annual and/or biennial flowers) are present, the fewer perennials there are and the lower the sustainability of the flower bed. This factor explains 19.27% of the variance.

### 2.5. The Flower Bed Sustainability Index (FBSI)

Various statistical descriptive measures were examined to investigate the Flower Bed Sustainability Index (Table 8).

**Figure 1:**
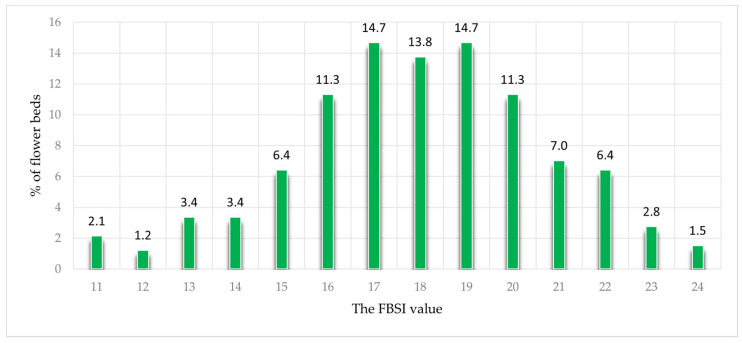
The distribution of the flower beds (in %) on the FBSI (*n* = 327).

The Flower Bed Sustainability Index recorded values ranging from 11 to 24, with the lowest score for seven flower beds and the highest for five (Figure 1).

**Figure 2:**
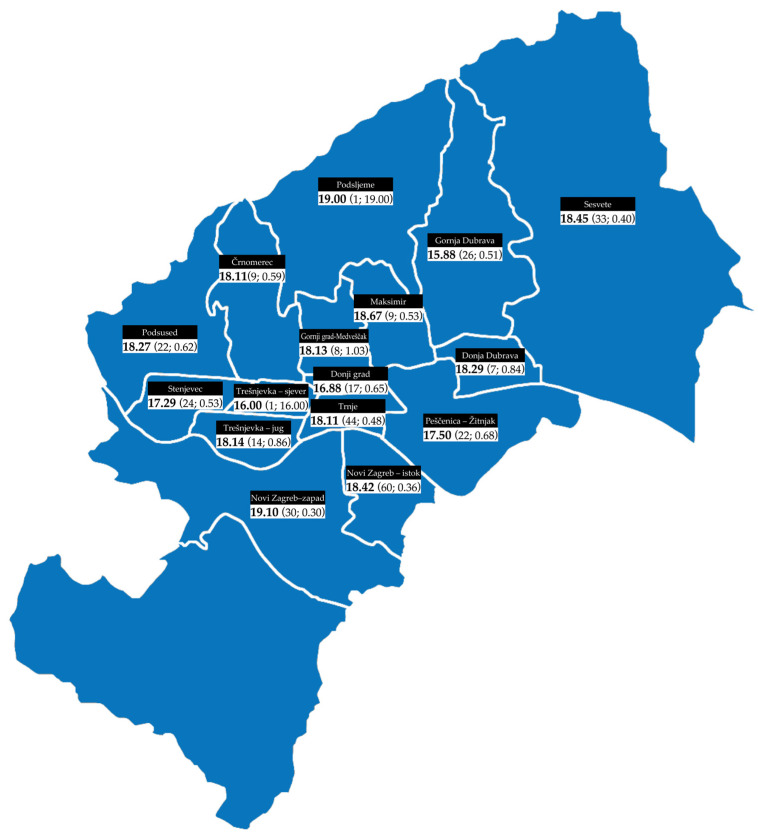
The distribution of the average value on the FBSI by city districts. The sample size (*n*) and the standard error (SE) are given in parentheses.

The lowest average value of the sustainability index was measured in the district of Gornja Dubrava, while the highest value was measured in Novi Zagreb—zapad (Figure 2).

**Figure 3:**
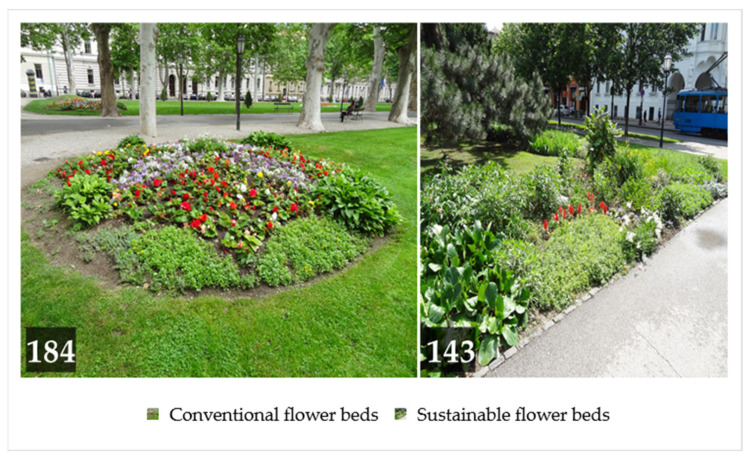
Typology of analyzed flower beds with perennials in the City of Zagreb (*n* = 327).

As there is not yet an adequate measurement tool to assess the sustainability level of a flower bed and consequently no criteria have been established to differentiate between conventional and sustainable flower beds, a provisional threshold of 18 points was set for the Flower Bed Sustainability Index. The number 18 represents the theoretical median value of the scale. Flower beds with a total score of 18 or less, which have fewer sustainability characteristics, were classified as conventional, while flower beds with a total score of more than 18, which have a higher level of sustainability, were classified as sustainable for the purposes of this study. Based on the mentioned typology, 56.3% of the analyzed flower beds were conventional and 43.7% sustainable (Figure 3).

The chi-square test was used to test the hypothesis that conventional flower beds with perennials predominate in the public green spaces of the City of Zagreb. Because a statistically significant difference was found between the theoretical and expected frequencies (χ^2^_(1)_ = 5.141, *p* = 0.023), the hypothesis was accepted (Table 9).

## 3. Materials and Methods

### 3.1. Study Area

The City of Zagreb is the capital and largest city of the Republic of Croatia. It is located in the continental part of the country (45.49° N, 15.59° E). According to the 2021 census, it has about 767,000 inhabitants, which is about 20% of the total population of Croatia [46]. In the context of urbanization, it is worth noting that this percentage has increased by 1.4% compared to the 2011 census (from 18.4% to 19.8%).

**Figure 4:**
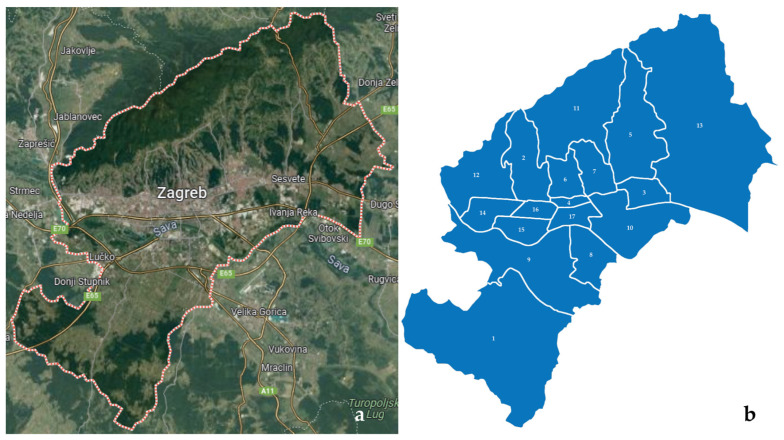
A Google Maps view of the City of Zagreb with (**a**) the marked border of the studied area and (**b**) the administrative division into city districts: 1—Brezovica, 2—Črnomerec, 3—Donja Dubrava, 4—Donji grad, 5—Gornja Dubrava, 6—Gornji grad—Medveščak, 7—Maksimir, 8—Novi Zagreb—istok, 9—Novi Zagreb—zapad, 10—Peščenica—Žitnjak, 11—Podsljeme, 12—Podsused—Vrapče, 13—Sesvete, 14—Stenjevec, 15—Trešnjevka—jug, 16—Trešnjevka—sjever, 17—Trnje.

The survey was conducted on the entire territory of the City of Zagreb (641 km^2^), which is divided into 17 city districts (Figure 4).

According to the Green Cadastre of the City of Zagreb, the total area of flower beds is 64,991 m^2^ and they are divided into three basic types depending on the plant material: flower beds with seasonal flowers, flower beds with perennials and flower beds with roses. There are a total of 640 flower beds with perennials, covering an area of 6745 m^2^. To determine which types of flower beds with perennials planted in the ground predominate in the City of Zagreb, the beds in containers and planters were excluded because they did not meet the basic requirements for sustainability. In addition, the beds without plant material were also excluded. The data on the location and total area of individual beds with perennials were taken from the Green Cadastre.

Information on the characteristics of perennial beds was collected through field studies over several years (from 2012 to 2019). The original intention was to analyze random samples of flower beds, but it was later decided to process all beds in order to eliminate the possibility of sampling error, i.e., the possibility of drawing false conclusions. More complex flower beds (e.g., those containing multiple species) were visited several times during the year to accurately identify any species present that might be less conspicuous during certain parts of the year. After the fieldwork, two control surveys were conducted in 2022 in two city districts that had been analyzed during the initial phase of the study. It was determined that there were no changes relevant to the study.

All data on flower bed characteristics were collected using the visual assessment method based on predefined indicators. The flower species were determined in consultation with the relevant scientific literature. Each flowerbed was documented photographically, and all relevant information was recorded into a specially created database.

### 3.2. Defining Criteria and Indicators

In order to evaluate the suitability of the species found in the flower beds in relation to environmental conditions, data from the State Hydrometeorological Institute (Zagreb-Maksimir measuring station) for the period from 2017 to 2021 were used. According to these data, the average annual temperature in the Zagreb area was 12.7 °C, while the absolute minimum and maximum were −14.6 and 37.7 °C, respectively. The average annual precipitation was 894.7 mm. For the evaluation of tolerance of perennials to low temperatures, the USDA (United States Department of Agriculture) classification (Plant Hardiness Zone) was used as a reference, according to which Zagreb is in zone 6b.

The characteristics of flower beds were analyzed using criteria and indicators, some of which were derived from other studies and recommendations [47,48,49] and modified for the needs of this study (Table 10).

### 3.3. Flower Bed Sustainability Index

An additive index was created based on nine selected indicators, with the hypothesis that all indicators are equally relevant for measuring the sustainability level of the flower bed. All indicators were assigned a three-point rating scale, i.e., each indicator was rated on a scale of three points (Table 11). The highest score (3) was assigned to characteristics that contribute most to the sustainability of the bed (correct selection of flower species, greater diversity of flower species, greater area coverage, extensive maintenance), while the lowest score (1) was assigned to characteristics that contribute least to sustainability (incorrect selection of flower species, lower species diversity, lower area coverage, intensive maintenance). The theoretical range of the constructed index was from a minimum of 9 to a maximum of 27 points.

Based on the points awarded for all indicators, an additive Flower Bed Sustainability Index (FBSI) was created by summing all points to determine the level of sustainability of each flower bed. A higher total FBSI score means that the bed is more sustainable, while a lower score means that it is more conventional. In this study, the terms “conventional” and “sustainable” flower beds were defined as ideal types.

To answer the question of which type of flower beds predominates in the public green spaces of the City of Zagreb, the flower beds were divided into two categories: conventional and sustainable. The median score of the Flower Bed Sustainability Index was used as a criterion for distinguishing between these two types of flower beds. All flower beds with a score equal or below the median score were treated as conventional flower beds, while those with a score greater than the median score (MDN = 18) were treated as sustainable flower beds.

### 3.4. Statistical Analysis

The data were analyzed using IBM SPSS Statistics (Version 27) software. The empirical data were analyzed using methods and procedures of descriptive, inferential (inductive) and multivariate statistics. In the context of descriptive statistics, the variables were analyzed using univariate techniques and appropriate descriptive statistical indicators (frequency distributions, percentage distributions, mean values, modal values, median values, standard deviation, skewness and kurtosis). The data were presented in tabular and graphical form. 

As part of the inferential statistical data analysis, the variables were analyzed using bivariate techniques, depending on the type of variables analyzed. When testing the association of nominal variables, the chi-square test was used taking into account the Yates correction for 2 × 2 tables and Fisher’s exact test when a cell with a theoretical frequency of less than 5 was observed in 2 × 2 tables. To test statistically significant differences between more than two categories of the independent variable on the dependent ordinal variable, the Kruskal–Wallis H test was used.

A multivariate analysis was used to examine the factor structure of a multiple-item instrument measuring flower bed characteristics. Principal component analysis was used, although ordinal variables were examined [52]. The factor solution was obtained using the GK criterion to extract statistically significant latent dimensions of flower bed characteristics and was subjected to an orthogonal transformation using the varimax rotation criterion. Considering that there is a strong theoretical and practical reason for retaining the maximum possible number of theoretically predicted dimensions [53], it was decided that we retain and interpret even those factors that saturate only two items. 

All statistical tests were performed at a 5% level of significance.

## 4. Discussion

The study included all flower beds with perennials in the City of Zagreb. Only those that were planted in the ground were analyzed, as those that were in planters or other containers did not meet one of the basic requirements for sustainability (contact with the soil). Based on predefined criteria and indicators, 327 beds with perennials were analyzed.

### 4.1. The Relationship between the Different Characteristics of the Flower Beds

The correct selection of plants is a basic requirement for a successful sustainable flower bed and therefore also for a green space. To achieve sustainability, a correctly selected plant must fulfill several criteria. It must be adapted to the conditions of the location, be able to be maintained with relatively little effort in terms of resources, water, nutrients and maintenance time, support biodiversity as much as possible, be attractive to people and more [44]. The correct selection of species is becoming increasingly important due to climate change, as plants are increasingly exposed to abiotic stress, which has a detrimental effect on plant growth and productivity [54,55]. Selecting plants that are tolerant to such stresses makes it possible to reduce maintenance costs, but also to maintain the esthetic value of green spaces. [56] and provide important ecosystem services [57,58,59]. A carefully planned selection of perennials ensures the longevity of the bed itself, and they require less maintenance [60], which was also proven in this study.

Research has shown that the coverage of the bed area is influenced by a good selection of species. The correct selection of perennials led to greater coverage of the bed and therefore less maintenance. The results are consistent with previous studies that have found a direct correlation between population density, i.e., land cover, and maintenance costs, as a higher density of plant material leads to lower maintenance costs [61]. The use of ornamental perennials and ground cover can reduce soil temperature and moisture as well as weed density while reducing maintenance and resource consumption, which contributes to sustainability [60,62]. The thinning of vegetation can make a negative impression on users, but it is assumed that the use of techniques that contribute to the rapid formation of ground cover would improve respondents’ visual preference for such places [63]. According to users, planting ground cover plants has a positive effect on the physical and chemical properties of the soil and increases the esthetic value of the green space [64]. Although the use of mulch, like surface cover, contributes to the sustainability of the flower bed by improving soil moisture, helping to maintain soil fertility, mitigating extreme temperatures in the root zone, reducing erosion, and reducing the occurrence of weeds [65], the presence of mulch was not an overly relevant factor in this study. In fact, mulch was only found on three flower beds in front of a commercial building in Sesvete that is not maintained by a municipal company. 

The diversity of flower species from different groups within the flower bed is one of the essential elements that determines its sustainability and influences the maintenance effort. In this study, it was found that the beds that contained seasonal species in addition to perennials were maintained more intensively, regardless of whether they were annuals or biennials, which means that the costs were higher. Although planting beds with perennials is more expensive than planting beds with seasonal flowers, they save a lot of time due to their longevity and resilience, thus reducing maintenance costs. For example, it is estimated that a mixed perennial planting with around 15 to 20 species takes around 5–8 min per m^2^ per year [66]. There are numerous studies showing that users prefer diversity and species richness [67,68], which is characteristic of a sustainable flower bed. It also promotes biodiversity and attracts pollinators, which is extremely important in today’s challenging times [69,70].

It was difficult to determine the degree of maintenance quite objectively. The visual assessment was based on the theoretical assumption that proper and regular care should result in excellent condition of the flower bed. In addition to the visual assessment, informal interviews were conducted with employees of the company responsible for the public green spaces of the City of Zagreb (Zrinjevac d.o.o.) and, where possible, with the local population. This provided interesting information on how a certain number of flower beds are planted and maintained by the residents living next to or in the immediate vicinity of the flower bed. Such a practice probably led to greater use of more diverse flower species from different groups, considering that such areas were perceived as private property by the direct users, as shown in other studies [15]. Such a perception of public space is understandable to a certain extent, as users stated that these green microsites were not adequately maintained until they started to take care of them.

Most of the intensively tended flower beds with perennials were located around the center of the city. This is understandable, as all the green spaces in this area are maintained in the same way in order to keep the esthetic impression at a high level. As the intensity of maintenance of flower beds with perennials decreases from the center of the city to the periphery, the city administration should consider a more active role for citizens in participating in projects related to flower beds. Previous studies show that citizens are willing to participate in such projects by taking part in the planting and maintenance, but not in the planning [15]. The residents who took part in the tree planting campaign in their neighborhood were more satisfied than those who did not. By participating in the project, they got to know each other better and were more satisfied with the appearance of their neighborhood. The least satisfied were those whose trees were planted by the investor without consulting them [71].

### 4.2. Flower Bed Sustainability Index

The value on the index indicates the degree of sustainability of the flower beds, i.e., their ability to survive in their current location with as little intervention as possible. The exact value that determines the precise boundary between a conventional and a sustainable bed is not and cannot be fixed, but for the purposes of categorization, it is defined as the median value of the theoretical range of the additive index. In reality, there is not too much difference between flower beds that have a value of 18 and those that have a value of 19 on the constructed index, as there is a continuum between these ideal types [21].

The research showed that conventional flower beds with perennials predominate in the city of Zagreb (56.3%), which confirmed the working hypothesis. Conventional flower beds are characterized by poor selection of species in relation to environmental conditions, low diversity of flower species, presence of flower species other than perennials (annual and/or biennial flower species), lower area coverage, lack of mulch and more intensive maintenance. The research results show that the use of perennials does not necessarily guarantee the sustainability of the flower bed. For its longevity and resilience, certain conditions must be met: the right choice of species based on the environmental conditions of the site, the inclusion of different species to enhance diversity that meets both visual and ecological criteria, ensuring the greatest possible coverage by planting lower species or planting ground covers and other measures that reduce the need for maintenance and thus the consumption of resources.

### 4.3. Limitations and Further Research

The instrument for measuring the characteristics of flower beds should be reviewed with a view to possible improvements through further studies. For example, the ordinal scales of the existing indicators may need to be extended to achieve more precise results. Furthermore, the inclusion of additional indicators that could influence the sustainability of flowerbeds should be considered. For example, whether native or invasive species have been used, whether the species used are attractive to pollinators, and the like. The Flower Bed Sustainability Index has certain limitations and there is room for improvement. This mainly concerns the evaluation of the existing scoring method, as not all indicators have the same influence on sustainability. For example, the right choice of plant material certainly has a greater influence on the sustainability of a flower bed than, for example, the presence of mulch. For this reason, further research is needed to evaluate and improve the method so that it is more accurate in assessing sustainability.

## 5. Conclusions

Perennials deserve a more prominent place in public green spaces, as their wider use is an important step towards greater sustainability in the urban landscape. To achieve the resilience and sustainability of a flower bed with perennials, it is necessary to use species that are fully adapted to the environmental conditions, but also take into account that these conditions are subject to correction due to climate change. Although they consume many resources, conventional beds with annual and biennial flower species will probably continue to occupy the most representative locations in cities due to their visual qualities. But perennials also have their trump cards, as they offer greater (bio)diversity, enrich the space with dynamic changes throughout the year through different flowering times and require far fewer resources, which automatically reduces maintenance costs.

## Figures and Tables

**Table 1:** Distribution of flower beds with perennials by city districts in the City of Zagreb (*n* = 327).

City District	f	%
Brezovica	0	0.00
Črnomerec	9	2.75
Donja Dubrava	7	2.14
Donji grad	17	5.20
Gornja Dubrava	26	7.95
Gornji grad—Medveščak	8	2.45
Maksimir	9	2.75
Novi Zagreb—istok	60	18.35
Novi Zagreb—zapad	30	9.17
Peščenica—Žitnjak	22	6.73
Podsljeme	1	0.31
Podsused—Vrapče	22	6.73
Sesvete	33	10.09
Stenjevec	24	7.34
Trešnjevka—jug	14	4.28
Trešnjevka—sjever	1	0.31
Trnje	44	13.46

Note: f, number of flower beds.

**Table 2:** Distribution of flower beds with perennials according to their characteristics by city districts in the City of Zagreb.

Indicator	Value	City Districts																f	%
		CD1	CD2	CD3	CD4	CD5	CD6	CD7	CD8	CD9	CD10	CD11	CD12	CD13	CD14	CD15	CD16		
C1I1 *	<50%	0	0	2	4	1	0	6	0	6	0	2	4	5	2	0	4	36	11.0
	50–99%	6	5	13	18	4	8	34	19	10	1	11	21	16	9	1	23	199	60.9
	100%	3	2	2	4	3	1	20	11	6	0	9	8	3	3	0	17	92	28.1
	Total	9	7	17	26	8	9	60	30	22	1	22	33	24	14	1	44	327	100.0
C2I1	1–3	3	1	1	6	5	1	15	7	16	0	10	3	3	3	1	19	94	28.7
	4–7	4	3	6	18	2	5	18	12	5	1	8	14	11	4	0	15	126	38.5
	>7	2	3	10	2	1	3	27	11	1	0	4	16	10	7	0	10	107	32.7
	Total	9	7	17	26	8	9	60	30	22	1	22	33	24	14	1	44	327	100.0
C2I2	40–100%	0	0	7	7	0	0	4	2	0	0	1	2	2	1	0	1	27	8.3
	10–39%	1	2	6	6	1	3	6	5	1	0	3	11	10	4	0	8	67	20.5
	<10%	8	5	4	13	7	6	50	23	21	1	18	20	12	9	1	35	233	71.3
	Total	9	7	17	26	8	9	60	30	22	1	22	33	24	14	1	44	327	100.0
C3I1	<50%	7	4	5	18	4	3	35	17	9	0	11	15	14	8	1	16	167	51.1
	50–79%	1	2	6	7	1	4	11	5	7	1	7	11	6	2	0	15	86	26.3
	80–100%	1	1	6	1	3	2	14	8	6	0	4	7	4	4	0	13	74	22.6
	Total	9	7	17	26	8	9	60	30	22	1	22	33	24	14	1	44	327	100.0
C3I2	<50%	7	6	10	24	4	4	42	18	11	0	15	20	16	8	0	20	205	62.7
	50–99%	2	1	7	2	4	5	12	7	8	1	7	13	8	5	0	21	103	31.5
	100%	0	0	0	0	0	0	6	5	3	0	0	0	0	1	1	3	19	5.8
	Total	9	7	17	26	8	9	60	30	22	1	22	33	24	14	1	44	327	100.0
C3I3	<50%	9	7	17	26	8	9	60	30	21	1	22	31	24	14	1	44	324	99.1
	50–99%	0	0	0	0	0	0	0	0	0	0	0	0	0	0	0	0	0	0.0
	100%	0	0	0	0	0	0	0	0	1	0	0	2	0	0	0	0	3	0.9
	Total	9	7	17	26	8	9	60	30	21	1	22	31	24	14	1	44	327	100.0
C4In1	Unmaintained	1	1	1	7	1	0	8	1	2	0	2	2	1	1	1	19	48	14.7
	Intensively maintained	0	0	14	2	1	0	2	1	4	0	0	0	0	0	0	1	25	7.6
	Extensively maintained	8	6	2	17	6	9	50	28	16	1	20	31	23	13	0	24	254	77.7
	Total	9	7	17	26	8	9	60	30	22	1	22	33	24	14	1	44	327	100.0

Note: * The results for indicators C1In2 and C1In3 are not shown because, due to maximum correlation, they have identical results as C1In1; CD1—Črnomerec; CD2—Donja Dubrava; CD3—Donji grad, CD4—Gornja Dubrava, CD5—Gornji grad—Medveščak; CD6—Maksimir; CD7—Novi Zagreb—istok; CD8—Novi Zagreb—zapad; CD9—Peščenica—Žitnjak; CD10—Podsljeme; CD11—Podsused—Vrapče; CD12—Sesvete, CD13—Stenjevec; CD14—Trešnjevka—jug, CD15—Trešnjevka—sjever; CD16—Trnje.

**Table 3:** The difference between the area of the flower bed and the maintenance level (*n* = 327).

Maintenance Level	*n*	Mean Rank	Statistical Significance of the Test Statistic
Unmaintained	48	153.11	Kruskal–Wallis H _(2)_ = 15.872 *** p = 0.000
Intensively maintained	25	236.04	
Extensively maintained	254	158.97	

Note: ***, significant at *p* < 0.001.

**Table 4:** The influence of the presence of other types of flowers on the maintenance level (*n* = 327).

Variable			Maintenance Level		χ^2^
			Extensive	Intensive	
The presence of other types of flowers	<10%	Observed	224	9	χ^2^_(1)_ * = 14.614 *** V = 0.224
		Expected	215.2	17.8	
	≥10%	Observed	78	16	
		Expected	86.8	7.2	

Note: χ^2^_(df)_ * = Pearson’s Chi-squared test with Yates’ continuity correction; V = Cramer’s V coefficient; ***, significant at *p* < 0.001.

**Table 5:** The influence of the correct selection of flower species on surface coverage (*n* = 327).

Variable			Coverage with Flower Species		χ^2^
			<50%	≥50%	
Properly selected flower species	<50%	Observed	27	9	χ^2^_(1)_ * = 8.225 *** V = 0.168
		Expected	18.4	17.6	
	≥50%	Observed	140	151	
		Expected	148.6	142.4	

Note: χ^2^_(df)_ * = Pearson’s Chi-squared test with Yates’ continuity correction; V = Cramer’s V coefficient; ***, significant at *p* < 0.001.

**Table 6:** The influence of the proper selection of flower species on the level of maintenance (*n* = 327).

Variable			Maintenance Level		χ^2^
			Extensive	Intensive	
Properly selected flower species	<50%	Observed	30	6	χ^2^_(1)_ * = 3.338 *** V = 0.119
		Expected	33.2	2.8	
	≥50%	Observed	272	19	
		Expected	268.8	22.2	

Note: χ^2^_(df)_ * = Pearson’s Chi-squared test with Yates’ continuity correction; V = Cramer’s V coefficient; ***, significant at *p* < 0.001.

**Table 7:** Principal component factor analysis of the attributes of flower beds.

Items	Factor Loadings		
	1	2	3
Proper selection based on resistance to low temperatures	0.99		
Proper selection based on light requirements	0.99		
Proper selection based on required precipitation	0.99		
Coverage with flower species		0.92	
Coverage with ground covers		0.91	
Presence of other flower species besides perennials			0.81
Number of flower species			−0.79
Eigenvalue	2.97	1.71	1.35
% of variance	42.45	24.46	19.27
Total explained variance	86.17%		

**Table 8:** Descriptive statistical measures of the Flower Bed Sustainability Index.

TR	ER	M	Median	SD	CV	Skewness	Kurtosis	KS
9–27	11–24	17.98	18.00	2.75	15.30	−0.24	−0.09	0.08 ***

Note: TR, theoretical range; ER, empirical range; M, arithmetic mean; SD, standard deviation; CV, coefficient of variability; KS, Kolmogorov—Smirnov test; ***, significant at *p* < 0.001.

**Table 9:** The result of testing the hypothesis that conventional flower beds with perennials predominate in the green spaces of the City of Zagreb (*n* = 327).

Type of Flower Bed	Observed Count	Expected	Residuals
Conventional	184	163.5	20.5
Sustainable	143	163.5	−20.5

**Table 10:** Criteria and indicators for analyzing the characteristics of flower beds with perennials.

Criterion	Indicator	Abbreviation	Description/Procedure
CRITERION 1. Adaptation of flower species to habitat conditions	Indicator 1. Proper selection based on resistance to low temperatures	C1I1	This indicator was operationalized as the percentage (%) of flower species within the flower bed that are properly selected based on their cold temperature resistance. The total percentage of properly selected flower species was divided into three ordinal categories: less than 50% properly selected, 50 to 99% properly selected, and 100% properly selected flower species. The USDA classification (Plant Hardiness Zone) was used as a reference classification, in which Zagreb is located in zone 6b, where the lowest temperatures range from −20.5 °C to −17.8 °C [50].
	Indicator 2. Proper selection based on light requirements	C1I2	This indicator was operationalized as the percentage (%) of flower species within the flower bed that are properly selected based on their light requirements. The total percentage of properly selected flower species was divided into three ordinal categories: less than 50% properly selected, 50 to 99% properly selected, and 100% properly selected flower species. The usual classification according to the light level required by the plants was used as a reference: full sun (more than 6 h of direct sun per day), partial shade (4 to 6 h of direct sun per day) and full shade (less than 4 h of direct sun per day). Based on the field analysis of the location of each flower bed (orientation and exposure), the suitability of the selected flower species was evaluated.
	Indicator 3. Proper selection based on required precipitation	C1I3	This indicator was operationalized as the percentage (%) of flower species within the flower bed that are properly selected based on their required amount of precipitation. The total percentage of properly selected flower species was divided into three ordinal categories: less than 50% properly selected, 50 to 99% properly selected, and 100% properly selected flower species. The following categorization according to water needs was used: low water needs, average water needs and high water needs. The relevant literature was used to classify the plants into the above categories and the appropriateness of the selection of species was assessed on this basis.
CRITERION 2. Diversity of flower species within the flower bed	Indicator 1. Number of flower species	C2I1	This indicator was operationalized as the total number of different flower species within the flower bed. The total number of flower species was divided into three ordinal categories: one to three flower species, four to seven flower species, and more than seven flower species.
	Indicator 2. Presence of other flower species besides perennials	C2I2	This indicator was operationalized as the percentage (%) of other flower species besides perennials within the flower bed. The total percentage of other flower species besides perennials was divided into three ordinal categories: 40–100%, 10–39%, and less than 10% of other flower species. Perennials were treated as the foundation of the flower bed, so annual and biennial flower species, as well as geophytes, were considered as other flower species.
CRITERION 3. Ground coverage	Indicator 1. Coverage with flower species	C3I1	This indicator was operationalized as the percentage (%) of the area covered by flower species within the total area of the flower bed. The portion of the area with flower species was divided into three ordinal categories: less than 50%, 50 to 79%, and 80–100% of the area covered with flower species. Coverage with flower species was determined by visual assessment of each bed.
	Indicator 2. Coverage with ground covers	C3I2	This indicator was operationalized as the percentage (%) of the flower bed area covered by ground covers. The total percentage of the area covered by ground covers was divided into three ordinal categories: less than 50%, 50 to 99%, and 100% of the area covered with ground covers. Coverage with ground covers was determined by visual assessment of each bed.
	Indicator 3. Coverage with mulch	C3I3	This indicator was operationalized as the percentage (%) of the total flower bed area covered by mulch. The total percentage of the area covered by mulch was divided into three ordinal categories: less than 50%, 50 to 99%, and 100% of the area covered with mulch. Coverage with mulch was determined by visual assessment of each bed.
CRITERION 4. Maintenance	Indicator 1. Maintenance level	C4I1	This indicator was operationalized by the intensity of flower bed maintenance. The intensity of flower bed maintenance was divided into three ordinal categories: unmaintained, intensively maintained, and extensively maintained flower beds. The reference classification was the National Habitat Classification of the Republic of Croatia [51]. The classification for Public Non-Productive Cultivated Green Spaces (I.8.1.) was applied. According to this classification, flower bed maintenance is divided into the following categories: A. Intensively Maintained Parks within Settlements (I.8.1.1.): Parks, gardens, and public green areas with trees, lawns, flower beds, and ornamental shrubs, intensively fertilized, irrigated, and maintained. Flowers are changed multiple times a year. The definition of this type at this level implies a spatial complex. B. Extensively Maintained Parks within Settlements (I.8.1.2.): Public park and green areas that are maintained once or twice a year, mainly focusing on grass mowing. The definition of this type at this level implies a spatial complex. In addition to the data on the level of maintenance that was received from the municipal company that maintains the city’s green spaces, an additional check was carried out through a visual assessment during the field inspection.

**Table 11:** Value of points for each indicator.

Indicator	Value	Points
Proper selection based on resistance to low temperatures	<50%	1
	50–99%	2
	100%	3
Proper selection based on light requirements	<50%	1
	50–99%	2
	100%	3
Proper selection based on required precipitation	<50%	1
	50–99%	2
	100%	3
Number of flower species	1–3	1
	4–7	2
	>7	3
Presence of other flower species besides perennials	40–100%	1
	10–39%	2
	<10%	3
Coverage with flower species	<50%	1
	50–79%	2
	80–100%	3
Coverage with ground covers	<50%	1
	50–99%	2
	100%	3
Coverage with mulch	<50%	1
	50–99%	2
	100%	3
Maintenance level	Unmaintained	1
	Intensively maintained	2
	Extensively maintained	3

## Data Availability

The data presented in this study are available on request from the corresponding author.
